# A Case of Amyand's Hernia

**DOI:** 10.7759/cureus.73305

**Published:** 2024-11-08

**Authors:** Nima Sadeghi, Jamie McDermott, Nazanin Kermanshahi, Ayman Anasi, Imtiaz Ahmed

**Affiliations:** 1 Medicine, Midwestern University Arizona College of Osteopathic Medicine, Glendale, USA; 2 Radiology, Tempe St. Luke's Hospital, Tempe, USA

**Keywords:** amyand, amyand hernia, appendix, hernia, vermiform, vermiform appendix

## Abstract

Inguinal hernias are the most prevalent type of abdominal wall hernia. While many cases are uncomplicated, some variant forms can pose a heightened risk of severe complications. We report the case of a 46-year-old male who arrived at the emergency department with a two-day history of diffuse abdominal pain, with an otherwise negative review of systems, an unremarkable medical and surgical history, and normal laboratory results. The physical examination revealed a palpable, non-reducible mass in the right groin, raising suspicion of an inguinal hernia. A CT scan of the abdomen and pelvis was conducted, confirming Amyand's hernia, characterized by the presence of the vermiform appendix within the hernia sac in the dilated right inguinal canal. Amyand's hernia is a rare and clinically challenging condition to diagnose because its symptoms are nonspecific and often resemble those of other inguinal hernias. Delayed diagnosis can heighten the risk of complications, including inflammation, infection, perforation, and acute appendicitis. Mortality rates for Amyand's hernias have been reported to be between 14% and 30%, primarily due to complications associated with infections, especially peritoneal sepsis. Prompt diagnosis and treatment of Amyand's hernia, usually involving surgical hernia repair and an appendectomy if appendicitis is present, are highly effective in preventing potentially life-threatening complications. Despite their rarity, the difficult nature of the diagnosis and the associated high mortality rate underscore the importance of considering Amyand's hernia as a serious differential diagnosis.

## Introduction

Hernias are a diverse group of conditions, with the majority occurring in the abdominal or groin regions. Among these, inguinal hernias are the most common type and can manifest at any age. A rare variant, Amyand's hernia, is characterized by the presence of the vermiform appendix within the hernia sac. Amyand's hernia occurs in approximately 1% of cases, with acute appendicitis complicating 0.08% to 0.13% and progressing in only about 0.1% of cases [1,2]. The preoperative diagnosis of Amyand's hernia is particularly challenging due to its variable clinical presentation. As a result, a definitive diagnosis is often made intraoperatively as an incidental finding. Typically, inguinal hernias are diagnosed based on clinical history and physical examination. Amyand's hernia frequently presents with right lower quadrant pain and an irreducible, tender mass in the inguinal or inguinoscrotal region, which may initially be mistaken for an incarcerated hernia [3]. The standard surgical treatment for Amyand's hernia includes both appendectomy and hernia repair [4,5]. However, there is debate over performing appendectomy when the appendix is not inflamed and using mesh in such cases. Some experts advise against prophylactic appendectomy if the appendix appears normal, while others recommend it to prevent potential reherniation or future appendicitis. The diagnostic challenges of Amyand's hernia arise from its nonspecific symptoms, which can easily mimic those of typical hernias. However, the current literature emphasizes that early recognition and appropriate imaging techniques, such as computed tomography (CT) scans, MRI, or ultrasound, are crucial for effective surgical planning and reducing postoperative complications [6]. Therefore, a high index of suspicion is essential for prompt diagnosis and timely intervention.

## Case presentation

**Figure 1:**
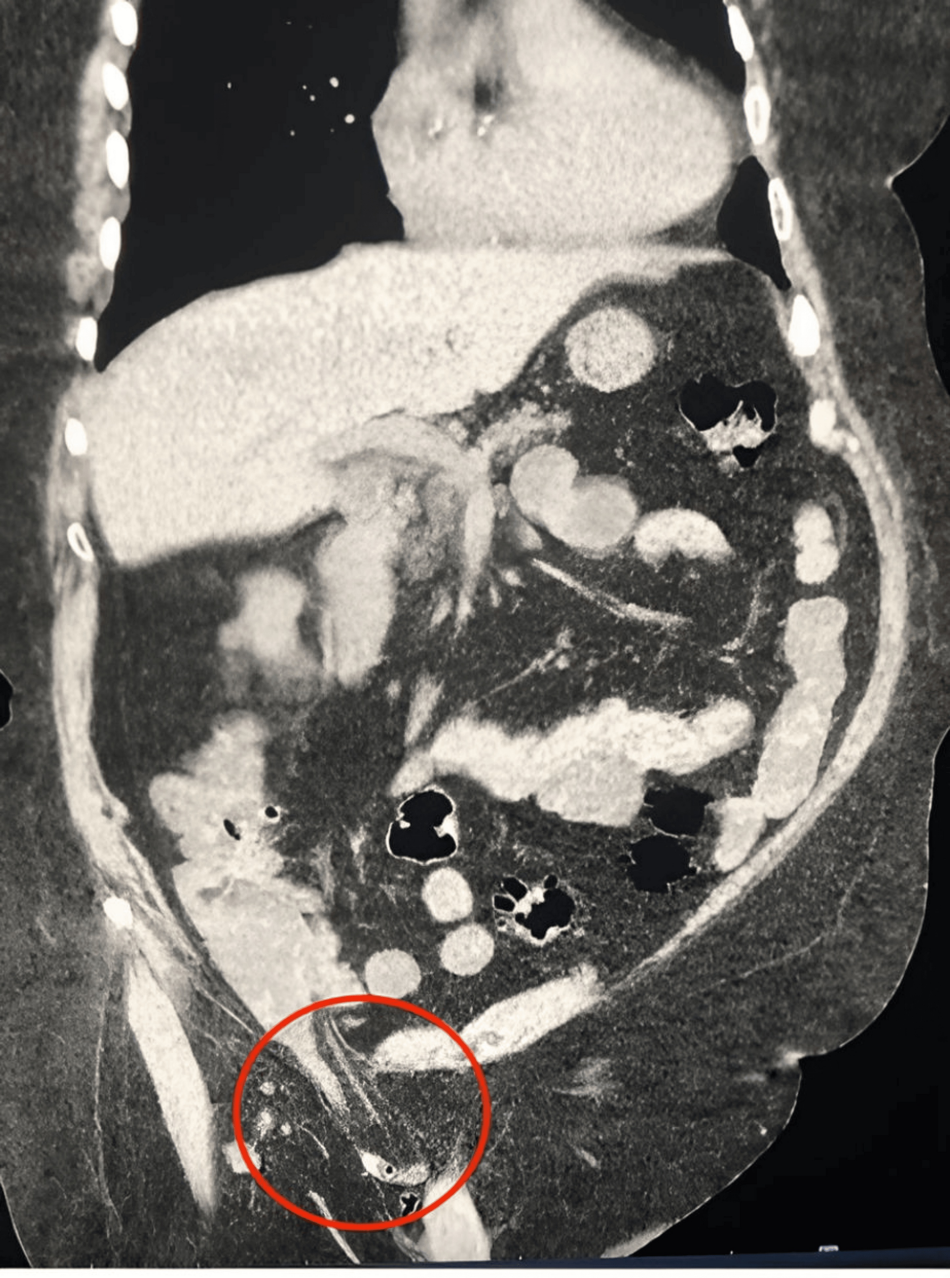
Coronal non-contrast CT of the abdomen showing a tubular structure arising from the cecum and mesenteric fat in the hernia sac (red circle).

**Figure 2:**
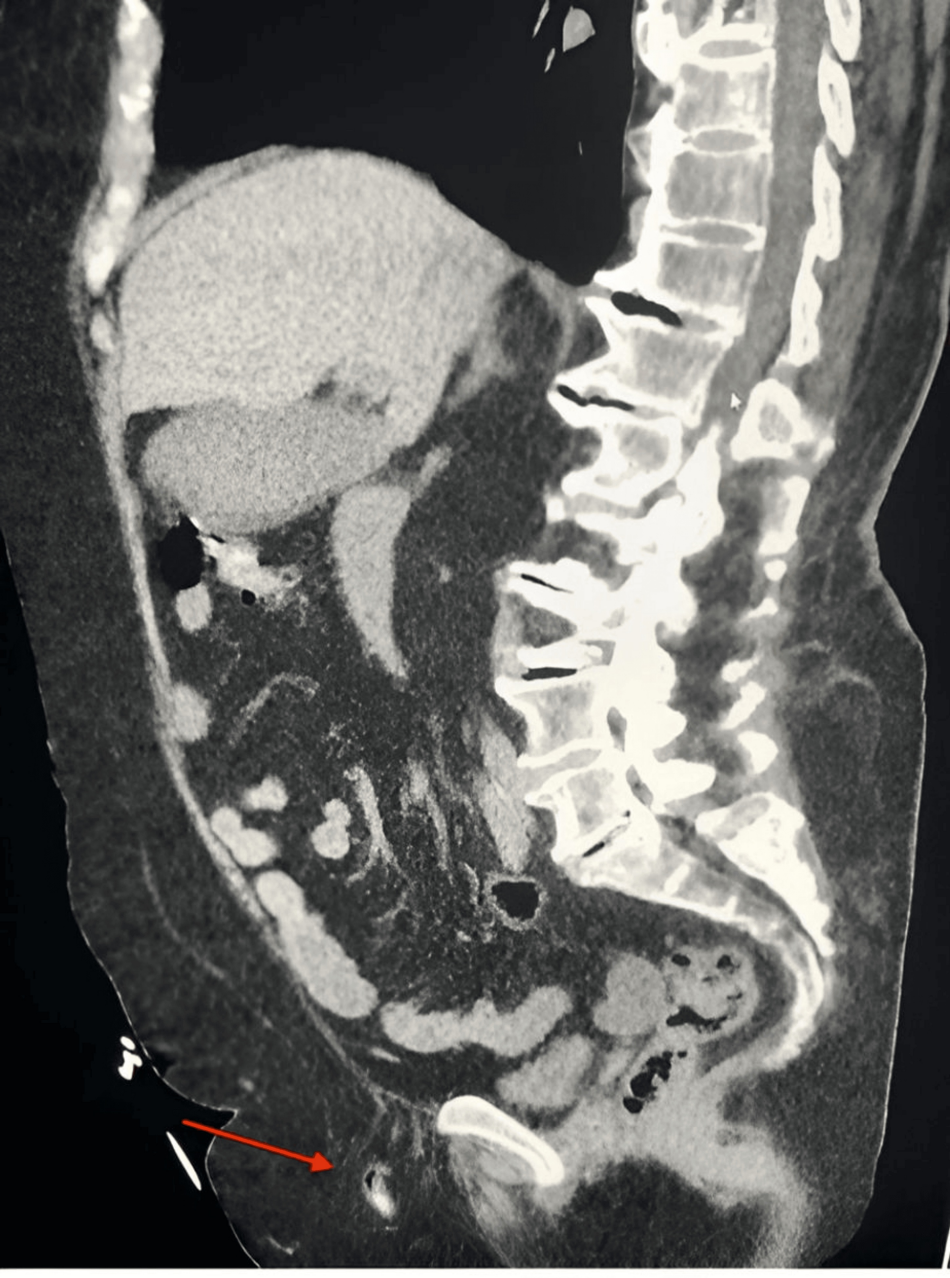
The sagittal CT of the abdomen shows a herniated appendix without any inflammation.

A 46-year-old male patient presented to the emergency department with a two-day history of diffuse abdominal pain. He denied fever, nausea, hematemesis, loss of appetite, dizziness, and urinary symptoms. His past medical and surgical history were unremarkable, and laboratory results were within normal limits. The physical examination revealed a non-reducible, right-sided palpable mass in the inguinal region, which increased in size with straining. The patient reported tenderness over the hernia site, particularly during attempts at reduction. An inguinal hernia was suspected, and an abdominal CT scan confirmed the presence of the vermiform appendix within the hernia sac (Figures 1, 2).

## Discussion

Amyand's hernia is a rare variant of inguinal hernia characterized by the presence of the appendix within the hernia sac, often leading to appendicitis. First described by Claudius Amyand in 1735, it typically presents with symptoms similar to those of a standard inguinal hernia, such as groin pain and a palpable bulge [7]. However, the co-occurrence of appendicitis can obscure the diagnosis, making recognition challenging. Due to nonspecific physical examination findings and a lack of distinctive clinical symptoms, Amyand's hernias are frequently identified incidentally during surgical procedures. Management typically involves surgical hernia repair, with an appendectomy performed if the appendix is found to be inflamed. Early diagnosis and intervention are crucial to prevent complications such as strangulation or rupture.

In this case, the patient presented with a two-day history of diffuse abdominal pain without any accompanying symptoms such as fever, nausea, hematemesis, anorexia, dizziness, or urinary complaints. Given the suspicion of an inguinal hernia, a CT scan was ordered to rule out strangulation or bowel perforation. The imaging revealed a blind-ending tubular structure connected to the cecum within the hernia sac, confirming the diagnosis of Amyand's hernia. While preoperative imaging is not routinely performed for inguinal hernias, the literature supports the use of ultrasound or CT scans in aiding decision-making and surgical planning [6].

There is currently no established gold standard for diagnosing Amyand's hernia. However, ultrasound is widely accessible, cost-effective, and provides real-time imaging without the ionizing radiation associated with CT, making it a safer option for use in pediatric and pregnant patients. The ultrasonographic criteria for diagnosing acute appendicitis include the identification of a blind-ending, noncompressible, non-peristaltic tubular structure larger than 6 mm, with appendiceal wall hyperemia observed via color Doppler [8]. Despite its advantages, the accuracy of ultrasound can be influenced by the operator's expertise and patient-specific factors, such as obesity or excessive bowel gas. Although ultrasound was not utilized in this case, it will be considered for future management of similar cases, particularly given the low incidence of Amyand's hernias and the need for further evidence regarding the role of ultrasound in preoperative diagnosis. The American College of Radiology (ACR) Appropriateness Criteria also finds an MRI abdomen with and without IV contrast appropriate for initial imaging if a groin hernia is suspected [9]. MRI offers significant advantages over CT and ultrasound for suspected inguinal hernias, including superior soft tissue contrast and multiplanar imaging, which enhance the accuracy of hernia visualization and characterization. It also avoids ionizing radiation, making it safer for patients requiring repeat imaging. However, the limited availability and longer scan durations of MRI render it less feasible in emergency settings where rapid diagnosis is essential [9,10].

## Conclusions

Amyand's hernia, although rare, should be included in the differential diagnosis of inguinal hernias due to its potential complications, including appendicitis and, in severe cases, sepsis. This case underscores the nonspecific nature of its clinical presentation, which can often resemble that of other types of hernias, thereby complicating preoperative diagnosis. Imaging techniques such as CT scans, MRI, or ultrasound, while not routinely employed, can be instrumental in the early detection and surgical planning of Amyand's hernias, thereby reducing the risk of complications such as strangulation or perforation. Although CT imaging was utilized in this case, ultrasound may serve as a non-invasive and patient-friendly diagnostic tool, offering minimal discomfort in future cases. MRI is preferred in a non-emergent outpatient setting. Given the low incidence and variable presentation of Amyand's hernia, it is crucial to maintain a high index of suspicion for timely diagnosis and effective management.
